# Purifying selection and birth-and-death evolution in the class II hydrophobin gene families of the ascomycete *Trichoderma/Hypocrea*

**DOI:** 10.1186/1471-2148-8-4

**Published:** 2008-01-10

**Authors:** Christian P Kubicek, Scott Baker, Christian Gamauf, Charles M Kenerley, Irina S Druzhinina

**Affiliations:** 1 Research Area of Gene Technology and Applied Biochemistry, Institute of Chemical Engineering, Vienna University of Technology, Getreidemarkt 9-1665, A-1060 Vienna, Austria; 2 Fungal Biotechnology Team, Chemical and Biological Process Development Group, Pacific Northwest National Laboratory, 902 Battelle Blvd., Richland, WA 99352, USA; 3 Department of Plant Pathology and Microbiology, Texas A&M University, College Station, TX 77843, USA

## Abstract

**Background:**

Hydrophobins are proteins containing eight conserved cysteine residues that occur uniquely in mycelial fungi. Their main function is to confer hydrophobicity to fungal surfaces in contact with air or during attachment of hyphae to hydrophobic surfaces of hosts, symbiotic partners or themselves resulting in morphogenetic signals. Based on their hydropathy patterns and solubility characteristics, hydrophobins are divided into two classes (I and II), the latter being found only in ascomycetes.

**Results:**

We have investigated the mechanisms driving the evolution of the class II hydrophobins in nine species of the mycoparasitic ascomycetous genus *Trichoderma/Hypocrea*, using three draft sequenced genomes (*H. jecorina = T. reesei, H. atroviridis = T. atroviride; H. virens = T. virens*) an additional 14,000 ESTs from six other Trichoderma spp. (*T. asperellum, H. lixii = T. harzianum, T. aggressivum *var. *europeae, T. longibrachiatum*, *T*. cf. *viride*). The former three contained six, ten and nine members, respectively. Ten is the highest number found in any ascomycete so far. All the hydrophobins we examined had the conserved four beta-strands/one helix structure, which is stabilized by four disulfide bonds. In addition, a small number of these hydrophobins (HFBs)contained an extended N-terminus rich in either proline and aspartate, or glycine-asparagine. Phylogenetic analysis reveals a mosaic of terminal clades containing duplicated genes and shows only three reasonably supported clades. Calculation of the ratio of differences in synonymous vs. non-synonymous nucleotide substitutions provides evidence for strong purifying selection (*K*_*S*_/*K*_*a *_>> 1). A genome database search for class II HFBs from other ascomycetes retrieved a much smaller number of hydrophobins (2–4) from each species, and most were from Sordariomycetes. A combined phylogeny of these sequences with those of *Trichoderma *showed that the *Trichoderma *HFBs mostly formed their own clades, whereas those of other Sordariomycetes occurred in shared clades.

**Conclusion:**

Our study shows that the genus *Trichoderma/Hypocrea *has a proliferated arsenal of class II hydrophobins which arose by birth-and-death evolution followed by purifying selection.

## Background

Hydrophobins are small proteins that are unique for mycelial fungi [1-4]. Their core structure consists of four beta strands, crosslinked by four disulfide bridges [5,6], creating a structure enabling the self-assembly at a hydrophilic/hydrophobic interface between the hydrophilic cell wall and a hydrophobic environment (such as air or the hydrophobic surface of living and non-living material). They are classified on the basis of chemical properties (hydrophobicity, solubility) into class I or class II hydrophobins [1], with class I hydrophobins having been identified in both ascomycetes and basidiomycetes, and class II hydrophobins having so far been only detected in ascomycetes [4]. Hydrophobins can fulfill a plethora of biological functions ranging from the formation of aerial structures, elicitation of morphogenesis and interaction with potential hosts or symbiotic partners [2,7-10].

In the primary sequence, the most important feature common to all hydrophobins is the characteristic pattern of eight Cys-residues, which gives rise to a common disulfide network [5,11]. Besides these conserved Cys-residues and similar hydropathy patterns, however, the hydrophobins share only a few conserved residues [4]. The poor amino acid sequence conservation of hydrophobins raises the question as to the evolutionary mechanism driving the rapid differentiation of hydrophobin gene sequences. Other genes involved in the response of organisms to their immediate environment have sometimes been shown to be driven by positive selection [12-16], so called "arms races" [17]. However, concerted evolution and birth-and-death evolution under strong purifying selection have also been reported [18-24].

The fungal genus *Trichoderma/Hypocrea *contains a large number of mycoparasitic species [25,26], and some of them (e.g. *H. lixii = T. harzianum*, *H. virens = T. virens*, *H. atroviridis = T. atroviride*, *T. asperellum*) are also commercially used as biological fungicides [27]. Interestingly, there are only a few reports describing the occurrence of hydrophobins in mycoparasitic strains of *Trichoderma/Hypocrea *[28-30] and a characterization of the function or biological role for each has not been determined. The class II hydrophobin genes *hfb1 *and *hfb2 *of the weakly mycoparasitic species *H. jecorina (T. reesei*) have been studied [31,32] and shown to serve different functions during vegetative development. Viterbo and Chet [33] recently showed that a class I hydrophobin from *T. asperellum *is involved in root colonization. Interestingly, this hydrophobin is the only class I hydrophobin identified in any *Trichoderma/Hypocrea sp. *so far.

The assignment of a function to individual members of large gene families like the hydrophobins is complicated by the possibility that several of them may have overlapping functions [34,35], which in turn is dependent on the selective pressures acting on the organism. Understanding the evolution of such genes and identifying stable clusters within the phylogeny may therefore help illustrate members with a potentially critical function.

## Results

### Protein structure of the *Trichoderma *class II hydrophobin proteins

In order to have a representative sample of class II HFBs from *Trichoderma/Hypocrea*, we first screened the available genome sequences of *H. jecorina*, *H. atroviridis *and *H. virens*, retrieving 6, 10 and 9 genes encoding class II proteins, respectively. Second, we searched NCBI and identified one HFB from *H. lixii *(= *T. harzianum*) strain T-22 (HL_4). We also included the hydrophobin-like protein QID3 from *H. lixii *strain CECT 2413 [36] in the analysis. A third hydrophobin – *srh1 *– from "*T. harzianum*" [28] was also included in this study, but as this strain had been misidentified and is in fact *H. atroviridis *[37] it turned out to be identical to HA_2a (see Table 1). Third, we screened the TrichoEST database [38] which – besides containing ESTs of *H. atroviridis *and *H. virens *– contains transcript sequences from additional five *Trichoderma/Hypocrea *species (*H. lixii, T. aggressivum var. europeae, T. longibrachiatum, T. stromaticum, T. asperellum *and *T*. cf. *viride*), resulting in 15 further HFBs. The identity of "*T*. cf. *viride"*was rechecked on the basis of ESTs for elongation factor 1 alpha (*tef1*) and RNA polymerase subunit B (*rpb2*) and determined to be closest to *T. koningiopsis*, and we will therefore name this strain *T*. cf. *koningiopsis *throughout this study. Our sample consisted of 42 class II HFBs from 9 different species of *Trichoderma*, covering sections *Longibrachiatum, Trichoderma *and *Pachybasium *(cf. Table 1), and thus consisting of a well distributed sample.

**Table 1:** *Trichoderma/Hypocrea *class II hydrophobin genes*

Trichoderma Section	*Trichoderma/Hypocrea *spp.	abbreviation	Scaffold	accession no.
Longibrachiatum	*H. jecorina*	HFB6	3:1189832–1190084	
	*H. jecorina*	HFB3	31:136511–136957	
	*H. jecorina*	HFB5	11:163081–163444	
	*H. jecorina*	HFB1	3:1189832–1190084	
	*H. jecorina*	HFB2	56:80872–81271	
	*H. jecorina*	HFB4	5:390006–390436	
				
	*T. longibrachiatum*	TL_1	L19T52P004R01376	[GenBank: AJ905782]
				
Trichoderma	*T. asperellum*	TA_1	L14T53P124R00732	[GenBank: AJ903054]
	*T. asperellum*	TA_2	L14T53P129R00833	[GenBank: AJ903147]
	*T. asperellum*	TA_3	L14T53P116R00634	[GenBank: AJ902899]
	*T. asperellum*	TA_4	L14T53P135R01371	[GenBank: AJ903666]
				
	*H. atroviridis*	HA_1b	1:719649–719242	[GenBank: EU053447]
	*H. atroviridis*	HA_1c	1:1159027–1159456	GenBank: EU053448] [GenBank: EU053449];
	*H. atroviridis*	HA_2a = SRH1	2:2051503–2051110	[GenBank: CAA72539]
	*H. atroviridis*	HA_2b	2:2503511–2503091	[GenBank: EU053450]
	*H. atroviridis*	HA_2c	2:3431445–3432224	[GenBank: EU053456]
	*H. atroviridis*	HA_5a	5:343144–342721	[GenBank: EU053451]
	*H. atroviridis*	HA_6a	6:627631–626945	[GenBank: EU053452]
	*H. atroviridis*	HA_6b	6:738694–738316	[GenBank: EU053453]
	*H. atroviridis*	HA_6c	6:1048590–1048979	[GenBank: EU053454]
	*H. atroviridis*	HA_22a	22:79408–78987	[GenBank: EU053455]
	*T. *cf. *Koningiopsis*	TCK_1	L21T78P020R01908	[GenBank: AJ909436]
	*T. *cf. *Koningiopsis*	TCK_2	L21T78P014R01340	[GenBank: EV554903]
	*T. *cf. *Koningiopsis*	TCK_3	L21T78P012R01144	[GenBank: EV554904]
				
Pachybasium	*H. virens*	HV_1a	1:2048115–2048517	[GenBank: EU053457]
	*H. virens*	HV_1b	1:2185848–2186274	[GenBank: EU053458]
	*H. virens*	HV_1c	1:1718557–1718103	[GenBank: EU053459]
	*H. virens*	HV_1d	1:909158–909955	[GenBank: EU053460]
	*H. virens*	HV_2a	2:562868–563256	[GenBank: EU053461]
	*H. virens*	HV_13a	13:955443–955019	[GenBank: EU053462]
	*H. virens*	HV_18a	18:175336–174917	[GenBank: EU053463]
	*H. virens*	HV_21a	21:388893–389498	[GenBank: EU053464]
	*H. virens*	HV_22a	22:232164–232586	[GenBank: EU053465]
				
	*H. lixii*	HL_1	L03T34P016R01491	[GenBank: AJ896766]
	*H. lixii*	HL_2	L02T34P126R11028	[GenBank: AJ896364]
	*H. lixii*	HL_3	L03T34P047R04364	[GenBank: AJ897108]
	*H. lixii*	QID3		[GenBank: X71913.1]
	*H. lixii*	HL_4		[GenBank: ABN64104]
				
	*T. aggressivum var. europeae*	TAE_1	L50TH2P009R00852	[GenBank: ES768856]
	*T. aggressivum var. europeae*	TAE_2	L50TH2P018R01702	[GenBank: ES768855]
	*T. aggressivum var. europeae*	TAE_3	L50TH2P001R00008	[GenBank: AJ904501]
				
	*T. stromaticum*	TS_1	L55TSTP002R00109	[GenBank: ES768859]

No accession numbers are given for *H. jecorina*, because its genome genome is available online; genome and EST data were obtained from the following strains: *H. jecorina *QM6a; *H. atroviridis *IMI 206040; *H. virens*, Gv 29-8; *T. asperellum *T53, *T. longibrachiatum *T52; *T. aggressivum *var. *europeae *CBS 453.93 *H. lixii *CECT 2413; *T*. cf. *koningiopsis *T78; *T. stromaticum *CBS 101875.

**Figure 1:**
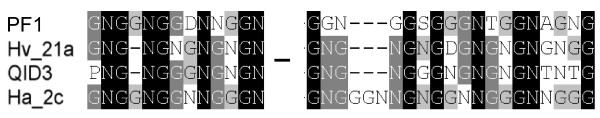
Amino acid alignment of a portion of the extended N-terminus of QID3, Ha_2c, Hv_21a and the *Passalora fulva *hydrophobin PF1 (for accession number see Table 4). The gap (indicated by a "-") is of different length in the four sequences and therefore not shown. Black background indicates absolutely conserved amino acids. Grey background indicates conservation in at least three of the four proteins.

Prediction of the encoded protein sequences showed that most of the predicted HFBs had the expected structure of 90 – 110 amino acids, which includes a 15–20 aa signal peptide, the 65 aa core structure displaying the eight cysteines which are predicted to have four 4 beta-strands and a single helix. However, five of them (one from *H. jecorina*; HFB6) and two each from *H. atroviridis *(Ta_2c and Ta_6a) and *H. virens *(Tv_1d and Tv_21a) contained also an additional N-terminal segment of 64 – 133 amino acids which was characteristically rich in P, G and N/D, and for which no secondary structure could be predicted with certainty. This extended N-terminus is similar to the one found in the *T. harzianum *pseudohydrophobin QID3, in which one conserved cysteine residue is replaced by a serine [36] and indicates that it is a general feature of a small group of *Trichoderma *class II hydrophobins. Moreover, within a subgroup of them, a characteristic G _x_N repeat was found to be conserved (Fig. 1).

**Figure 2:**
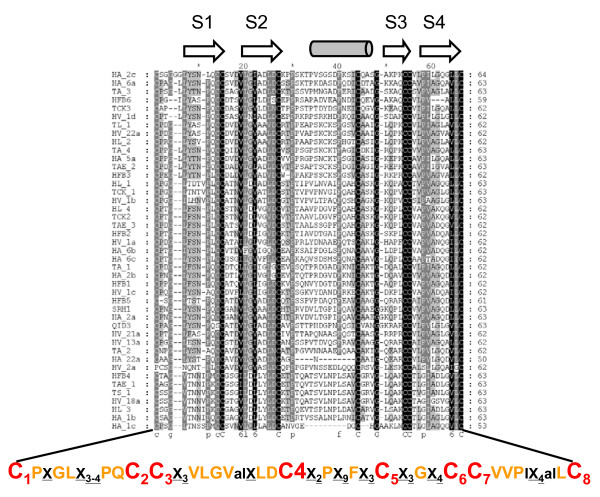
Amino acid alignment of the class II hydrophobins of *Trichoderma/Hypocrea *used in this study. The aa sequences were trimmed to show only the area from the first to the eight cysteine. Absolutely conserved aa's are within a black background, and functionally conserved aa's highlighted in grey. The symbols and letters over the alignment show the position of the four beta-strands (S1–S4) and the single helix (indicated by a horizontal cylinder). The sequence below the alignment proposes an updated consensus sequence for the *Trichoderma/Hypocrea *class II HFBs, as derived from this study: therein, the cysteines are in red and numbered in order of their appearance in the sequence; X denotes any amino acid, and the subscript the number of them; "al" denotes any aliphatic, hydrophobic amino acid (A, V, L, I,)

Fig. 2 shows the aa-alignment of the *Trichoderma *class II hydrophobins. Most of the conserved residues are located in the four β-strands and around the conserved cysteines. The aa's forming the α-helix, in contrast, showed very little conservation. Two of the HFBs, i.e. Ta_1c and Ta_22a, had part of the helical domain deleted and likely represent pseudogenes. This hypothesis is also supported by our lack of finding transcripts of these two HFBs in a total of 40.000 ESTs from different stages of *H. atroviridis *development (C.P. Kubicek and S.E. Baker, unpublished data). However they otherwise showed the conserved amino acid sequence pattern typical for class II HFBs, and were therefore retained in the analysis. The alignment led to the identification of 32 aa (of a total of 64 aa) that were functionally conserved (Fig. 2).

### Genomic organisation of the *Trichoderma *hydrophobin genes

In order to identify the mechanisms acting on the evolution of the *Trichoderma *hydrophobin genes, we first looked at their genomic organisation and exon structures. The six *T. reesei *hydrophobins are located on five different, large scaffolds, and even the two which are located on the same scaffold (*hfb1 *and *hfb6*) are separated by over 700,000 bp, and are therefore unlinked. Similar, although several of the *hfb *genes of *H. atroviridis *and *H. virens *were located on the same scaffold, they were separated by over 100,000 bp. In order to analyse whether any of these loci would be syntenic across *Trichoderma *species, we subjected each of the *H. jecorina hfb *genes plus 5 kb of its up- and downstream nt-sequences to a TBLAST search in the genome sequences of *H. virens *and *H. atroviridis*. The loci flanking the six *hfb *genes in *H. jecorina *did not flank any of the *H. virens *and *H. atroviridis *genes found by this analysis, although the flanking genes alone were sometimes located in the same region on a different scaffold (data not shown). Together, these data suggest that the genome regions containing hydrophobin gene loci have undergone extensive recombination during their evolution.

### Intron/Exon structure of the *Trichoderma *hydrophobin genes

All of the chromosomal *hfb *genes of *H. jecorina, H. virens *and *H. atroviridis *contain two introns, which are very similar although not identical in size, and are positionally conserved. Interestingly, the length of the second exon encodes the aa sequence which folds exactly into the single α-helix of the hydrophobin and its third beta-sheet (cf. [5]) is absolutely conserved in all genes.

### Phylogeny of the *Trichoderma *class II hydrophobin proteins

**Figure 3:**
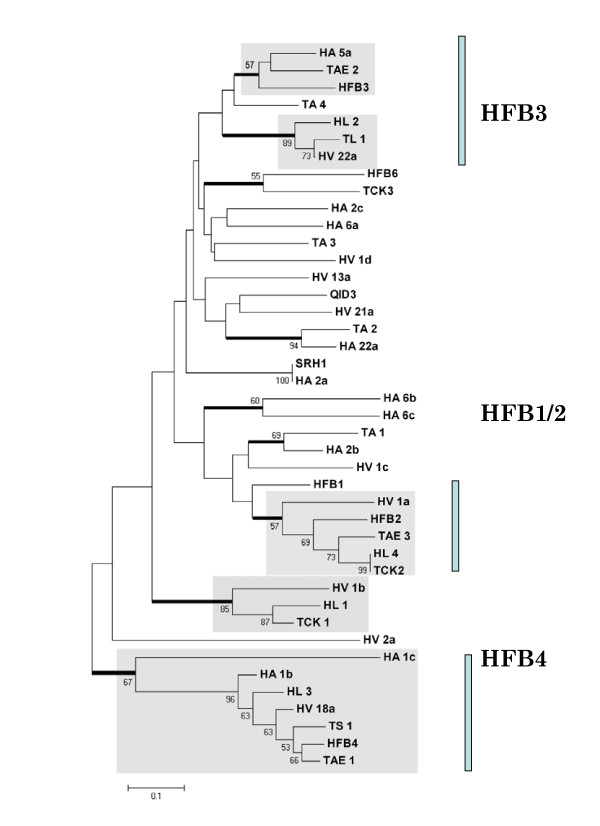
Phylogenetic analysis of *Trichoderma *class II hydrophobins. The already published proteins from *H. jecorina *(HFB1-6) is marked in red. Branchess receiving significant support (> 50% bootstrap values) are indicated with a fat line. Significantly supported clades, which contain hydrophobins from at least 3 different species, are underplayed in grey. The vertical bars mark the clades termed HFB1/2, HFB3 and HFB4.

A phylogeny, based on neighbour joining of the amino acid sequence area from C1 to C8_+1_, is given in Fig. 3. It is conspicuous that the internal branches of the tree are essentially unresolved, and statistically supported clades only occur in terminal branches. Bayesian analysis of the same dataset produced essentially consistent results (data not shown). Five strongly supported clades contain HFBs from more than two *Trichoderma *species, i.e. the clades containing *H. jecorina *HFB1/HFB2; the clade containing TCK1; the clade containing TL1; the clade containing *H. jecorina *HFB3; and the clade containing *H. jecorina *HFB4. We considered it possible that the poor resolution of the internal tree branches could be due to the lack of conservation in the α-helix, the phylogeny was also performed on an alignment from which the aa's forming the helix had been removed. However, this did not improve clade stability (data not shown).

### Evidence for gene duplications within the *Trichoderma *HFB proteins

One feature, which became obvious from the phylogenetic analysis and which is unaffected by the low internal branching support, is the high number of paralogous proteins. Examples for this are: Ha_1b and Ha_1c; Ha_6a and Ha_2c; Hv_21a and Hv_22a; TCK1 and TCK2; Ha_6b and Ha_6c; and HFB1 and HFB2. Most of these twins form a terminal branch, or are connected by a single node, indicating that they arose by gene duplication. The *Trichoderma *class II hydrophobins thus display a significant pattern of gene duplications in their evolutionary history.

### Nucleotide Sequence Divergence of the hydrophobin genes

**Figure 4:**
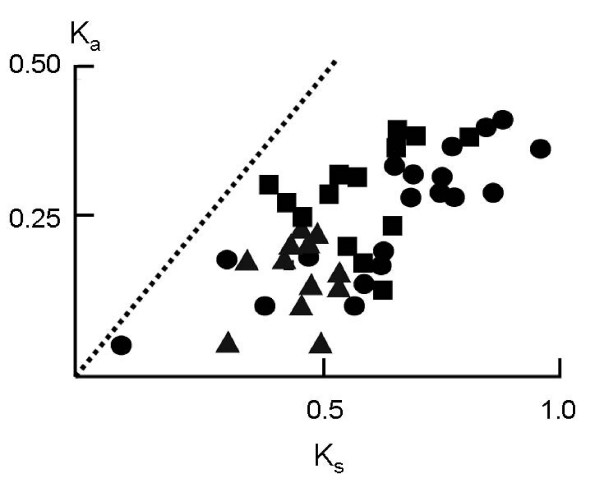
Plot of one-by-one comparisons of *K*_*a *_vs. *K*_*s *_for individual hydrophobin genes within clade "HFB1/2" (●), "HFB3" (■) and "HFB4" (▲) (for explanation of clades see Fig. 6). The dotted line indicates the position of *K*_*a*_/*K*_*s *_= 1.

In order to obtain an insight in the mechanisms driving the evolution of the *Trichoderma hfb *genes, we investigated their nucleotide sequences. Introns were thereby excluded. Bayesian analysis, based on an alignment of the nucleotide sequences starting from the triplet encoding the first cysteine (C1) and ending with that of the eighth cysteine (C8), produced a phylogenetic tree which basically showed the same clade structure as the tree based on aa alignment, only with poorer support of some of the interior branches (data not shown). We investigated the nucleotide phylogeny split decomposition [39,40], a method depicting the shortest pathway by linking sequences, rather than forcing them into a bifurcating tree. The resulting tree is shown in Fig. 4, demonstrating indeed a dense network in the interior branches. The highest probability for a tree like structure was obtained with branch leading to the "Hfb4" clade (cf. Fig. 3). Since such networks may be the consequence of recombination, we applied the Phi-test, implemented in SplitsTree. However, the results of the phi-test favour the rejection of the null hypothesis of recombination (p = 0.888). Consistent results were obtained by using a sliding window approach in TOPALi (data not shown). We therefore conclude that the interior network in the tree revealed by the split decomposition method is due to a loss of genes (and thus branching information) and not recombination.

The lack of recombination within the *hfb *genes, together with the observation of gene duplications suggests that the *Trichoderma *hydrophobins undergo purifying selection without concerted evolution. In such a case, the member genes would evolve independently and display a birth-and-death evolution [18]. This model of evolution assumes that new genes are created by repeated gene duplication and that some of the duplicate genes are maintained in the genome for a long time whereas others are deleted or become non-functional [18-20]. Thus nucleotide sequence differences between genes will primarily occur at synonymous sites, thus resulting in *K*_*S *_>> *K*_*a*_.

**Figure 5:**
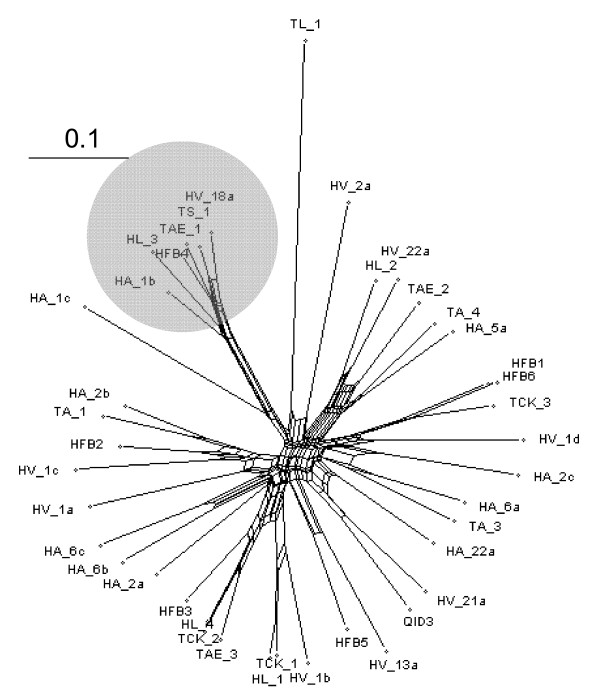
Phylogenetic tree of the nucleotide sequences of *Trichoderma/Hypocrea *class II hydrophobin genes by the split decomposition method. The "HFB4" clade, whose branch shows the least reticulate network, is highlightened in grey.

We therefore separately tested the total number of synonymous and nonsynonymous sites, as well as the number of nonsynonymous substitutions per nonsynonymous site (*K*_*a*_), and the number of synonymous -or silent-substitutions per synonymous -or silent-site (*K*_*s*_) [41], both within the total gene exon sequence as well as within the two major exons separately (Table 2). In total, the number of differences at nonsynonymous sites strongly exceeded those at synonymous sites. However, the number of nucleotide substitutions at synonymous sites (*K*_*S*_) was significantly higher than the number of substitutions in nonsynonymous sites (*K*_*a*_) in the complete gene and in exon 1, and slightly higher in exon 2. Plotting *K*_*S *_vs. *K*_*a *_for members of selected clades, which had obtained support in the aa-phylogeny (cf. Fig. 3) showed that the *K*_*S *_values for some gene-to-gene comparisons are very high (up to 1.0) and have apparently reached the saturation level [21]. Interestingly, different clades showed different maximal *K*_*a *_values, the "HFB4" clade thereby displaying the lowest numbers (Fig. 5).

**Table 2:** Nucleotide diversity of the *Trichoderma/Hypocrea *class II hydrophobin genes

	all three exons	exon 1	exon 2
no of sites	234	101	35
variable sites	95	52	30
no syn sites	21.39	13.65	8.52
no nonsyn sites	65.61	40.35	24.48
Eta, no of mutations	218	123	76
GC content	0.585	0.598	0.567
Pi nt diversity	0.4259	0.38773 ± 0.0164	0.42532 ± 0.0139
Theta per site (from Eta)	0.5	0.49	0.49
Tajima's D	-0.51296	-0.74828	-0.44716
K_S_	0.613	0.608	0.594
K_A_	0.367	0.312	0.376

### The *Trichoderma *HFB proteins form unique clades within the ascomycetous hydrophobins

**Figure 6:**
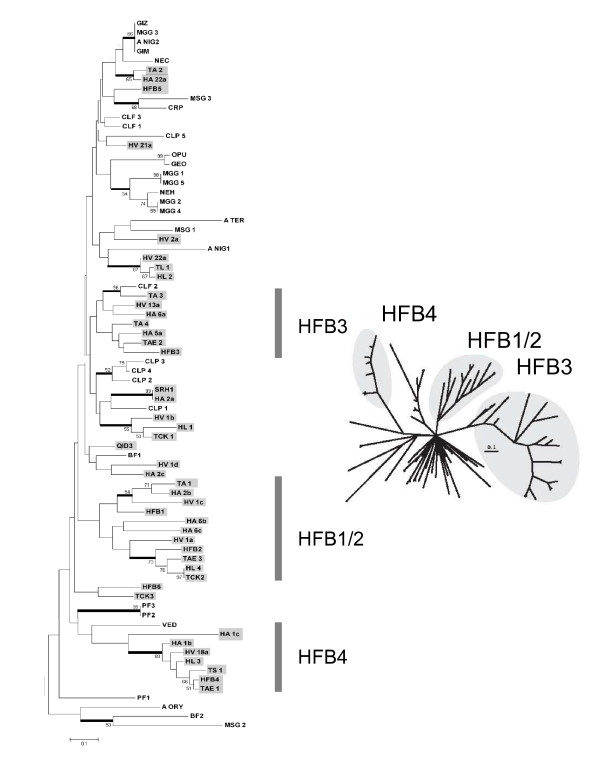
NJ analysis of amino acid sequences of class II hydrophobins from *Trichoderma *and other ascomycetes. Conditions and design of figure are similar as for Fig. 3. Accession numbers and/or genome database entries for the non-*Trichoderma *sequences are provided in Table 2. The inset on the right bottom shows the topology of an unrooted Bayesian tree (the three *Trichoderma *clades being highlightened in grey).

The findings of gene duplication and apparently non-functional genes raised the question as whether a similar pattern of paralogous genes would be found also in other ascomycetes, and whether their members would help to stabilize the clades formed by the *Trichoderma *class II HFBs. To this end, we screened the available genome databases of other ascomycetes by TBLAST, using members of each of the *Trichoderma *HFB clades as a query (see Materials and Methods). In addition, we searched the NCBI database for previously described class II HFBs. The result was interesting in so far as most other ascomycetes for which a draft genome sequence is available contain a much smaller number of hydrophobin genes than *Trichoderma*, *M. grisea *being richest with 5 proteins (Table 3). While we cannot absolutely rule out that some HFB-encoding genes slipped through our analysis because of low similarity to query sequences, this would at the same time imply that they do not belong to any of the clades established for *Trichoderma*, and thus not affect the purpose of this study (but see also the Discussion below). The respective 26 sequences were aligned with those of *Trichoderma *and used for a phylogenetic analysis. The result, shown in Fig. 6, illustrates two points: first, most of the proteins from *Trichoderma *formed their own clades. Second, most of hydrophobins from the other 26 fungi formed clades which received only poor support, which is best seen by the star phylogeny obtained by Bayesian analysis (inset in Fig. 6). Third, gene duplications were evident for those fungi, for which more than two genes had been found, e.g. *M. grisea *MGG1 and5, and MGG2 and 4; for *P. fulva *PF2 and 3.

**Table 3:** Class II hydrophobin genes from other ascomycetes used in this study

subphyllum	family	species	Protein name	Accession number *
Leotiomycetes	Sclerotiniaceae	*Botryotinia fuckeliana*	BF1	[*B. fuckeliana *genome database: BC1G_03994.19]
		*Botryotinia fuckeliana*	BF2	[*B. fuckeliana *genome database: BC1G_01012.1 ]
				
Eurotiomycetes	Trichocomaceae	*Aspergillus oryzae*	A_ORY	[GenBank: AAO16870.1]
		*Aspergillus terreus*	A_TER	[GenBank: XM_001213908]
		*Aspergillus niger*	A_NIG1	[GenBank: XM_001394993]
		*Aspergillus niger*	A_NIG2	[GenBank: AAN76355.1]
				
Dothiodiomycetes	Mycosphaerellaceae	*Mycosphaerella graminicola*	MSG3	[*M. graminicola *genome database: FGENESH2_PG.C_SCAFFOLD_8000534]
		*Mycosphaerella graminicola*	MSG2	[*M. graminicola *genome database: FGENESH2_PG.C_SCAFFOLD_2000556]
		*Mycosphaerella graminicola*	MSG1	[*M. graminicola *genome database: FGENESH2_PG.C_SCAFFOLD_11000390]
		*Passalora fulva*	PF3	[GenBank: CAC27408.1]
		*Passalora fulva*	PF1	[GenBank: CAC27407.1]
		*Passalora fulva*	PF2	[GenBank: CAB39312.1]
				
Sordariomycetes	Nectriaceae	*Gibberella moniliformis*	GIM	[GenBank: AY158024]
		*Gibberella zeae*	GIZ	[GenBank: FG01831.1]
		*Nectria haematococca*	NEH	[*N. haematococca *genome database: e_gw.1.52.181.1]
				
	Phyllachorales	*Verticillium dahliae*	VED	[GenBank: AAY89101]
				
	Cryphonectriaceae	*Cryphonectria parasitica*	CRP	[GenBank: L09559]
				
	Clavicipitaceae	*Claviceps fusiformis*	CLF	[GenBank: CAB61236.1]
		*Claviceps purpurea*	CLP	[GenBank: CAD10781.1]
				
	Ophiostomataceae	*Ophiostoma ulmi*	OPU	[GenBank: Z800849
				
	Magnaporthaceae	*Magnaporthe grisea*	MGG4	[GenBank: XM 364289]
		*Magnaporthe grisea*	MGG1	[GenBank: AF126872]
		*Magnaporthe grisea*	MGG2	[GenBank: XM_001522792]
		*Magnaporthe grisea*	MGG3	[GenBank: XM_382007]
		*Magnaporthe grisea*	MGG5	[GenBank: XM_364289]
				
	Sordariaceae	*Neurospora crassa*	NEC	[GenBank: XM_954189]

### Expression analysis of the "HFB4"-clade hydrophobin genes from *H. atroviridis*

**Figure 7:**
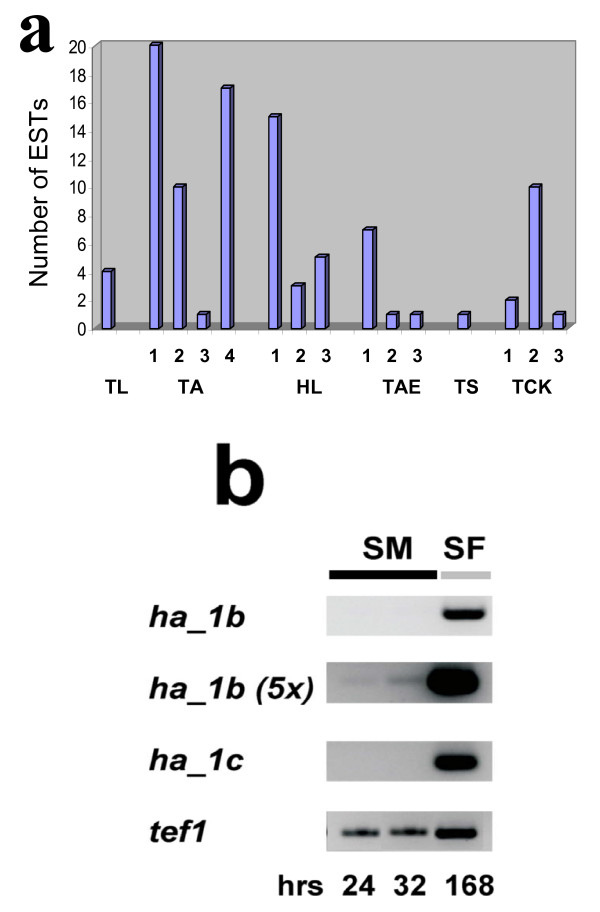
Gene expression of the *Trichoderma/Hypocrea *hydrophobins. (A) Number of ESTs found for the respective *hfb *genes during screening of the TrichoEST database. Species are abbreviated as follows: TL, *T. longibrachiatum*; TA, *T. asperellum*, HL, *H. lixii*; TAE, *T. aggressivum *var. *europeae*; TS, *T. stromaticum*; TCK, *T*. cf. *koningiopsis*. Strain numbers are given in the legend to Table 2. Individual hydrophobin genes are indicated by their respective numbers (cf. Table 1), and no number is given for species in which only a single hydrophobin gene has been detected. (B) Expression of the *H. atroviridis *gene members of the "HFB4" clade. SM, submerged cultivation; SF, surface cultivation. "5×" indicates that the 5-fold amount of PCR product had been loaded onto the gel.

In order to obtain an estimate of the relative expression of the various *Trichoderma/Hypocrea *hydrophobin genes, we first compared the numbers of ESTs in the TrichoEST database (Fig. 7a). The transcripts, which were most abundant, were from HFBs clustering in different clades, indicating that there is no cluster or group which is preferentially strongly expressed. In order to investigate the expression of members of the largest supported clade (the "HFB4" clade), we grew *H. atroviridis *in submerged and surface culture and examined the expression of the Ha_1b- and Ha_1c-encoding genes by RT_PCR (Fig. 7b). The results show that both genes are indeed expressed, and thus both duplicated copies are probably still functional, but are found only during growth on solid and not in submerged medium.

## Discussion

In this paper, we have investigated the evolutionary processes which give rise to the biodiversity of the fungal class II hydrophobin genes and proteins. This class of hydrophobins has so far been reported to be restricted to Ascomycetes only. Results from this study, however, suggest that the distribution of these genes may be even more restricted, i.e. the majority of the members of this class was actually found in the Sordariomycetes, and only few were found in Leotiomycetes and Eurotiomycetes. This picture may however be biased by the fact that Sordariomycetes are overrepresented sequenced genomes, and the six genes which we retrieved from the two species of Dothidiomycetes (*Passalora, Mycosphaerella*) suggest that this subphylum may also be rich in class II hydrophobins. We cannot completely rule out that the low number retrieved for Leotio- and Eurotiomycetes could be due to a failure to identify these genes by BLAST search. However, our approach also identified several class I hydrophobin genes from all these fungi (data not shown), and we would therefore assume that our screening was broad enough to identify all class II genes. Also, our results are in agreement with the results of manual annotation of several fungal genomes (*Aspergillus *spp., *M. grisea*, *N. crassa*, *G. zeae*, *N. haematococcae*). Therefore, while it is possible that a potential HFB encoding gene has been overlooked, our data indicate that while most species contained only 1 or 2 genes (e.g. *Gibberella*, *Nectria, Botryotinia, Aspergillus *spp.), species of *Trichoderma/Hypocrea *clearly exceed this with their gene number (i.e. 6 genes in *H. jecorina*, 9 in *H. virens *and 10 in *H. atroviridis*). The reason for this remains obscure: neither the morphology of the hyphae, the conidia or of the perithecium of *Hypocrea/Trichoderma *show microscopic differences which may necessitate new or multiple hydrophobins to support these structures. What differentiates this fungal genus from others, however, is its mycoparasitic and necrotrophic lifestyle [25]. While completely speculative at this moment, it is nevertheless possible that a versatile arsenal of class II hydrophobins may help the fungus to attach to the hyphae of a broad range of asco- and basidiomycetes. An amplified spectrum of genes has also been found for the chitinases of *H. jecorina*, which undoubtedly also aid to its mycoparasitic abilities [42]. With the availability of the hydrophobin gene sequences now in hand for two strongly mycoparasitic species – *H. atroviridis *and *H. virens *– this work lays a strong phylogenetic foundation to investigate this possibility by means of respective knock-out strains and expression analysis.

The results from this paper show that the class II hydrophobin genes of *Trichoderm/Hypocrea *contain a high number of duplicated genes, and at least two cases of pseudogenes. This suggests that the class II hydrophobins evolve by a death-and-birth mechanism [22], a term which has been created for a process in which genes undergo gene duplications, resulting in the maintenance of some of the copies for a considerable period of time whereas other copies are rapidly lost or converted to pseudogenes. Our data render the operation of concerted evolution unlikely, because of the high sequence divergence and also by the absence of recombination at the hydrophobin loci. (in concerted evolution, member genes evolve together as a unit by mechanisms such as gene conversion or unequal crossing-over). The fact that most of the duplicated genes occupy terminal branches in phylogenetic trees and that only few obvious pseudogenes were found, indicates that the rate of evolution of the class II hydrophobins in *Trichoderma *is relatively fast. This rapid evolution, and equally rapid loss of some genes is also reflected in the findings that the clades leading to the *hfb *genes in *Trichoderma *seldom contain members of other fungi, and their evolution thus took place after formation of the genus *Trichoderma*.

In addition, the numbers of synonymous differences of nucleotide sequences between genes from the same species are very large and frequently close to the saturation level. This high level of synonymous differences further supports the claim of a birth-and-death evolution at the DNA level, and supports the long time persistence of these genes in the genome. On the other hand, genes from different species (e.g. *H. atroviridis *and *H. virens*) but belonging to the same phylogenetic clade are highly similar (cf. Fig. 3). Such a long-term conservation of amino acid sequence is best explained by strong purifying selection. Interestingly, and in contrast to Rajashekar et al. [43], we found only a few individual cases where the *K*_*a*_/*K*_s _ratio was >1 and would reveal a history of accelerated evolution. If such a period of accelerated evolution occurred, most of the gene duplicates from this time apparently have not been maintained and the *Trichoderma/Hypocrea *hydrophobin genes characterized in this study are therefore mostly of recent origin.

The present study also expands our knowledge on the structure of class II hydrophobins. While most of them are small, compact proteins, which contain little other structures than the four beta-sheets and the single helix [5,6], we have detected several proteins which display a long extended N-terminus (ENT). With respect to class II HFBs, such structures have so far only been found in *H. jecorina *HFB6 [44], and in the pseudohydrophobin QID3 [36]. Interestingly, an ENT was recently also identified in the class I HFB Hum3 from *Ustilago mayidis *[45]. Lora et al. [46] hypothesized that the ENT of QID3 mediates cell wall binding because it resembles a module which is also present in plant bimolecular proteins [47-49]. Interestingly, our work reveals that there are at least two types of these ENTs: a major one, typified by *H. virens *HV_21a, *H. atroviridis *HA_2c, *H. lixii *QID3, and also in *P. fulva *HCF6, and in the spacers between the hydrophobin units in the multipartite genes of *C. paspali *and *C. fusii*, which are characterized by a conserved repeat of glycine and asparagines; and second type, shown by e.g. *H. atroviridis *HA_6a, *H. virens *HV_1d, and *H. jecorina *HFB6, in which the repeated motif is replaced by several PG/PD repeats, a P-rich stretch or a D-rich stretch, respectively. These proteins did not cluster together, indicating that these proteins do not show a common ancestry of the cysteine-containing core domain. Among the proteins with this terminus, one (HV_13a) is intriguing as its ENT is very short, which gives rise to the speculation that this extended N-terminus may arise by segment duplication. Support for this hypothesis would also come from the multipartite hydrophobins found in *Claviceps *spp. [50,51], wherein paralogous hydrophobin gene copies are connected by P, G and N-rich loops, and which may have been trapped in the stage of gene duplications at the extended N-terminus before recombining individual copies into new loci. It is thereby intriguing to observe the similarity of the nucleotide sequence of the "GN" repeat (GGTAAT) to that found to act as a recombination hot-spot in *Penicillium chrysogenum *(TGTAA [A/T]; [52]). Therefore, the occurrence of the *Claviceps *multipartite hydrophobins would be due to multiplication of some of the class II hydrophobins by tandem duplication [53,54], for which these sequences could act as recombination targets.

Nevertheless, it may still be likely that these ENTs are not only evolutionary artefacts: the [GN] repeats are reminiscent of *S. cerevisiae *Ure2p, a regulator of nitrogen catabolism, which can become transformed into a prion form by polymerization into filaments [55]. These filaments have an amyloid fibril backbone formed by an N-rich sequence which form a parallel superpleated beta-structure. The prion domain is thereby divided into nine seven-residue segments, each with a four-residue strand and a three-residue turn, that zig-zag in a planar serpentine arrangement, the interior of the filament being stabilized by H-bond networks generated by the stacking of N side chains. Interestingly, hydrophobins themselves are known to form amyloid-like structures [56-58], and we consider it therefore possible that the ENTs form defined structures which additionally contribute to the structural rigidity of the hydrophobins.

During phylogenetic analysis of the amino acid sequence, most hydrophobins from *Trichoderma/Hypocrea *did not group into strongly supported clades. However, a few exceptions were noted, notably the clades containing *H. jecorina *HFB1, HFB2 and HFB4, respectively. Clade "HFB4" is intriguing as its members – in contrast to HFB1 and HFB2 [31] were not expressed in submerged culture but only found in surface cultivation. This clade may thus contain hydrophobins relevant for hyphal growth. Unfortunately, the differences in aa-sequence with that of the other *Trichoderma *hydrophobins do not provide a clear clue as to the understanding of its function. One notable change is the substitution of the phenylalanine residue in the middle of the single helix, which is otherwise conserved in the class II hydrophobins from all other fungi (with the exception of *A. niger *and *V. lecanii *which contain an L and M, respectively), by a leucine, which may give rise to weaker hydrophobic interaction within the protein (hydrophobicity index F = 2.24; L = 1.99). However, Linder et al. [3] speculated that the aromatic ring of F_39 _is inserted between two Pro-residues (P_11 _and P_50_) from the two β hairpin structures into the protein and may serve to stabilize the fold through hydrogen bonds. Interestingly, members of the "HFB 4 clade" consistently have P_11 _replaced by an A which may hydrophobically interact with this L. In addition, the first beta-strand contains a conserved motif of two asparagines which provide it with a positive charge. Hydropathy plots show that members of the "HFB4 clade" have almost no hydrophobicity in the area between aa20 and aa40, and their helix is in contrast positively charged. While the consequence of these changes on the structure and function is however unclear our phylogenetic analysis sets the stage for future functional studies that may include transcript and gene deletion analysis.

## Conclusion

Summarizing, this study offers a model of evolution which gave rise to the biodiversity of class II hydrophobins. The more than 70 members identified in this study enabled us to delineate the consensus for both essential aa sequence parts as well as for the tolerance to aa modifications to these small compact proteins. Our phylogenetic analysis will inform future functional genomic studies aimed at determination of more specific functions for each of the *Trichoderma/Hypocrea *class II hydrophobins. In view of the strong potential of the hydrophobins in "white" biotechnology, this information may offer their further improvement by molecular evolution [59-61].

## Methods

### Conditions of fungal growth

*Hypocrea atroviridis *(anamorph: *Trichoderma atroviride*) strain P1 (ATCC 74058) was maintained on potato dextrose agar (PDA) (Difco, Franklin Lakes, NJ, USA). The following medium was used for its cultivation (g·l^-1^; [41]): D-glucose, 10; peptone, 0.35; Tween 80, 0.175; KH_2_PO_4_, 0.68; K_2_HPO_4_, 0.87; (NH_4_)_2_SO_4_, 1.7; KCl, 0.2; CaCl_2_, 0.2; MgSO_4_·7H_2_O, 0.2; FeSO_4_·7H_2_O, 0.02; ZnSO_4_·7H_2_O, 0.02; MnSO_4_·7H_2_O, 0.02. The fungus was grown either in 1 L shake-flasks containing 200 ml of the medium at 25°C (250 RPM) or on plates (in this case the medium was solidified by the addition of 15 g·l^-1 ^agar).

### Analysis of hydrophobin gene expression

Mycelia were withdrawn at selected time points as indicated and total RNA isolated by the method of Chomzynski and Sacchi [62]. The RNA extract was treated with DNAse I (Fermentas, St. Leon-Rot, Germany) and purified using the RNeasy MinElute Cleanup Kit (Qiagen, Hilden, Germany). The purified RNA was reverse transcribed using the RevertAid H Minus First Strand cDNA Synthesis Kit (Fermentas) with the oligo(dT)_18 _primer supplied by the manufacturer.

Appropriate aliquots of the cDNA were used for amplification by PCR utilising the GoTaq™ system (Promega, Madison, WI, USA). The assays contained 2.5 mM MgCl_2 _and 0.4 μM of the forward and reverse primer each (Table 4). The primers were designed in such a way that they aligned to different exons of the *hfb *genes to detect a possible contamination with genomic DNA. The amplification protocol consisted of an initial 1 min denaturation step at 95°C, followed by 28 cycles of denaturation (1 min at 95°C), annealing (1 min, see Tab. 1 for temperatures) and elongation (1 min at 72°C) and a final elongation step (7 min at 72°C). 40 μl of each assay were separated on 2% agarose containing 0.5 μg·ml^-1 ^ethidium bromide. Expression of the elongation factor 1-alpha (*tef1*) gene [63] served as a loading control. For negative controls, the DNAse digestion, cDNA synthesis and the PCR were repeated without addition of the reverse transcriptase, in which case no amplicons were detected, thus confirming that the detected bands indeed result from cDNA synthesised from RNA (data not shown).

**Table 4:** Primers used for RT-PCR

Target	Primer name	5' -> 3' sequence	T_2 _[°C]	Size [bp]
*ha_1 b*	hfb4RTfw	CTGCTTCTGAGGTCGTCGAG	59.0	244
	hfb4RTrv	GGAAGAGCATCCTGGCAC		
*ha-1c*	hfb5RTfw	CTCTTTACATTGGGCCTCG	55.5	208
	hfb5RTrv	CAAGAGTGCAGCAATTGAGC		
*tef1*	tef1RTFw	GTACTGGTGAGTTCGAGGCTG	59	350
	tef1RTRv	GGGCTCGATGGAGTCGATG		

### *In silico *screening for hydrophobin sequences from *Trichoderma *and other fungi

To obtain the *Trichoderma *class II hydrophobins, we used the sequences of the six class II hydrophobins of *H. jecorina *[44] as a tool to retrieve genes encoding proteins with similarity from the genome sequence databases of *Hypocrea virens *and *Hypocrea atroviridis *and the TrichoEST EST database which includes EST sequences from 9 different *Trichoderma *spp. [38] using TBLAST (protein vs translated nucleotide). In the case of duplicates, the genomic sequence was given preference. Table 1 summarizes the genes, proteins, accession numbers or locations of the sequences thereby retrieved.

To obtain class II hydrophobin genes from other ascomycetes, the genes compiled by Linder et al. [3] were used as a starting point for a TBLAST search of the NCBI data base, using the filtering option turned off, and sequences which had not yet been included by Linder et al. [3] were retrieved. Apart of genes deposited from specific research, this database contains genome sequences from *Neurospora crassa*, *Magnaporthe grisea, Gibberella zeae*, and several *Aspergillus *spp. In addition, the same procedure was used to mine the genome databases of *Nectria cinnabarina *[64], *Mycophaerella graminicola *[65] and *Botryotinia fuckeliana *(= *Botrytis cinerea*, [66]), fungi whose sequences have not yet been included in the NCBI database. Genes thereby retrieved were then themselves used as a query in BLAST search as described above, and the procedure repeated until no new gene/protein was detected. The amino acid sequences of the retrieved proteins were aligned, and class I hydrophobins (identified according to the criteria described by Linder et al. [3]; e.g. by the difference in the number of amino acids between the conserved cysteins and their hydropathy profile) removed.

### Phylogenetic analysis

DNA and protein sequences were visually aligned using Genedoc 2.6 [67]. Phylogenetic trees were constructed by the neighbor-joining (NJ) method [68], using the computer program MEGA, Version 4.0 [69], and by Bayesian analysis (MrBayes v3.0B4 program). The model of evolution and prior settings for individual loci was GTR + I + Γ. Metropolis-coupled Markov chain Monte Carlo (MCMCMC) sampling was performed with four incrementally heated chains that were simultaneously run for 1 and 3 millions of generations. To check for potentially poor mixing of MCMCMC, each analysis was repeated three times. The convergence of MCMCMC was monitored by examining the value of the marginal likelihood through generations. Convergence of substitution rate and rate heterogeneity model parameters was also checked. Bayesian posterior probabilities (PP) were obtained from the 50% majority rule consensus of trees sampled every 100 generations after removing the 500 first trees using the "burn" command. PP values lower then 0.95 were not considered significant.

### Test for recombination

Two different procedures were applied to detect recombination by comparing adjacent sequence windows and to detect significant departures from a single phylogenetic history within the same alignment. First, we used difference of sums of squares (DSS, [70]) to compare the fit of genetic distance matrices for two adjacent windows to the same tree topology to produce the DSS statistic, the significance of which was determined through parametric bootstrapping in TOPALi [71].

In addition we tested whether recombination would be apparent from the phylogeny of hydrophobin genes. This was done by the split decomposition method in SplitsTree, version 2.4 [40], using pairwise distances under the Kimura 3-ST model [72]. This method visualizes recombination events by depicting the shortest pathway linking sequences, rather than forcing them into a bifurcating tree [39,40].

### Tests for evolutionary mechanisms

To test the fit of the sequences to the model of neutral evolution, the *D *test statistic proposed by Tajima and Nei [73] was computed with the DnaSP program [74]. To this end, the Genedoc alignment was exported as a PHYLIP interleaved format. Only coding sequences, after removal of the preprosequence-encoding nt areas, were used for the analyses. Introns were removed from chromosomal nt-sequences by comparison with available cDNA (EST) sequences, or by relying on prediction of consensus splicing sites [75,76]. In the case of the multipartite hydrophobins from *Claviceps *spp. [50,51], each hydrophobin-encoding nt-area was treated as a separate entity. The extent of nucleotide divergence was estimated by using the uncorrected *p *distance [77]. The proportions of synonymous (were calculated *p*_S_) and nonsynonymous (*p*_N_) differences per site by the modified Nei-Gojobori method implemented in DNASp [12].

## Competing interests

The author(s) declares that there are no competing interests.

## Authors' contributions

CPK designed the study, performed the sequence alignments and evolutionary analyses and drafted the manuscript. SB coordinated the *H. atroviridis *genome sequence analysis and annotated the *H. atroviridis *and *H. virens *sequences. CG carried out the expression studies. CMK coordinate the *H. virens *genome sequence analysis and provided the *H. virens *sequence. ISD designed and performed the phylogenetic analyses. All authors read and approved the final manuscript.
